# Content Analysis of Emoji and Emoticon Use in Clinical Texting Systems

**DOI:** 10.1001/jamanetworkopen.2023.18140

**Published:** 2023-06-13

**Authors:** Colin M. E. Halverson, Claire E. Donnelly, Michael Weiner, Joy L. Lee

**Affiliations:** 1 Center for Bioethics, Indiana University School of Medicine, Indianapolis; 2 Department of Medicine, Indiana University School of Medicine, Indianapolis; 3 Department of Anthropology, Indiana University, Indianapolis; 4 Charles Warren Fairbanks Center for Medical Ethics, Indianapolis, Indiana; 5 Regenstrief Institute, Indianapolis, Indiana; 6 Center for Health Information and Communication, US Department of Veterans Affairs, Veterans Health Administration, Health Services Research and Development Service CIN 13-416, Richard L. Roudebush VA Medical Center, Indianapolis

## Abstract

**Importance:**

Emoji and emoticons are quickly becoming an omnipresent feature of virtual communication. As health care systems increasingly adopt clinical texting applications, it is critical to understand how clinicians use these ideograms with colleagues and how it may affect their interactions.

**Objective:**

To evaluate the functions that emoji and emoticons serve in clinical text messages.

**Design, Setting, and Participants:**

This qualitative study’s content analysis of clinical text messages from a secure clinical messaging platform was conducted to assess the communicative function of emoji and emoticons. The analysis included messages sent by hospitalists to other health care clinicians. A subset of a random 1% sample of all message threads, which included at least 1 emoji or emoticon, on a clinical texting system used by a large, Midwestern US hospital from July 2020 until March 2021 were analyzed. A total of 80 hospitalists participated in the candidate threads.

**Main Outcomes:**

Whether and what kind of emoji or emoticon was deployed in each reviewed thread was tabulated by the study team. The communicative function of each emoji and emoticon was assessed according to a prespecified coding scheme.

**Results:**

A total of 80 hospitalists (49 [61%] male; 30 [37%] Asian, 5 [6%] Black or African American, 2 [3%] Hispanic or Latinx, 42 [53%] White; of 41 with age data, 13 [32%] aged 25-34 years, 19 [46%] aged 35-44 years) participated in the 1319 candidate threads. Within the sample of 1319 threads, 7% of threads (155 unique messages) contained at least 1 emoji or emoticon. The majority (94 [61%]) functioned emotively, that is, conveyed the internal state of the sender, and 49 (32%) served to open, maintain, or close communication. No evidence was identified that they caused confusion or were seen as inappropriate.

**Conclusions and Relevance:**

This qualitative study found that when clinicians use emoji and emoticons in secure clinical texting systems, these symbols function primarily to convey new and interactionally salient information. These results suggest that concerns about the professionalism of emoji and emoticon use may be unwarranted.

## Key Points

**Question:**

How do clinicians use emoji and emoticons in professional communication with colleagues?

**Findings:**

In this qualitative study of 1319 clinical text message threads, 7% of threads contained an emoji or emoticon. The emoji and emoticons primarily added emotive content (61%) and/or served to open, maintain, or close channels of communication (32%).

**Meaning:**

This study’s results suggest that emoji and emoticons are used primarily to convey new and interactionally salient information, and concerns about professionalism in their use may therefore be unwarranted.

## Teaser

This qualitative study investigates the functions that emoji and emoticons serve in clinical text messages.

## Introduction

Increasingly, health care communication is occurring virtually via text-based messaging applications on smartphones and similar devices. With the proliferation of tools like the electronic health record (EHR), patient portals, and clinical texting systems (CTS), clinicians are interacting more frequently with patients and team members using virtual modalities. This shift in practice raises questions and concerns about the utility and appropriateness in clinical settings of certain features unique to electronic communication, such as emoticons and emoji. Emoticons are representations of faces constructed only with text characters from a traditional keyboard layout, for example, :( or ;-). Emoji, on the other hand, are typically 12×12-pixel images of everything from faces (😊) to eggplants (🍆) to exclamation marks (❗), which are inserted into digital text. Emoji were first introduced in the 1990s in Japan, but they were not added to the Unicode Standard until 2009, at which point they became available to an international community of users. Today, emoji are deployed with great frequency across many platforms, having in large part supplanted emoticons.^1^ It has been reported that 92% of the online population use emoji.^2^ While studies on communication have found successful integration of emoji and emoticons—which as a group we refer to as ideograms—in both informal and professional contexts,^3,4^ their use in health care settings has primarily been examined in patient-clinician interactions.^5,6,7,8,9^ The appropriate usage of ideograms and their impact on intraprofessional communication remain unclear.

While researchers have advocated for building consensus on the use of ideograms by clinicians and patients^10^ and suggested (albeit lightheartedly) that their use may affect how they are perceived by colleagues in health care,^11,12^ few have examined actual use by practicing medical professionals. Despite this gap in our knowledge, opportunities for deploying ideograms in clinical practice are ever increasing: CTS applications often make emoji available within their own or supported keyboards, and inserting emoji may also be possible in portal messages with patients. The increasing adoption of such computer-mediated communication in place of traditional alphanumeric pagers intensifies both the use of and the need for consensus on ideograms and their role in health care contexts.

To that end, the purpose of this study was to analyze data from a CTS to examine whether and how clinicians use emoji and emoticons. Additionally, we investigated implications for professionalism in clinical team communication. To our knowledge, this study represents the first to examine the use of these ideograms in actual communication between medical colleagues.

## Methods

### Setting

This study was conducted using data drawn from Diagnotes, a dedicated, proprietary, third-party CTS used by several large health care systems, including the Midwestern academic medical center at which our study was conducted. Two years prior to data collection, our study site had adopted a CTS to replace pagers as the primary communication tool between clinicians. Study team members included a doctorally trained linguistic anthropologist, a health services researcher with expertise in electronic health communication, a clinical informaticist, and a research specialist working in clinical communication. Our study was reviewed and approved by the Indiana University Institutional Review Board with waiver of informed consent because the study was not deemed human participants research. The use of the data for research and aggregate reporting was also approved by the medical center and covered by the privacy policy of Diagnotes. We followed the Consolidated Criteria for Reporting Qualitative Research (COREQ) reporting guideline.

### Keyboard

Diagnotes users have access to a traditional ASCII keyboard as well as the full set of Unicode emoji in the form of a supplementary keyboard palette, including faces, abstract symbols, and medical paraphernalia. At the time of data collection, there were nearly 2000 stem emoji in the Unicode Standard. As with many smartphone keyboards, emoji in our study’s CTS were organized by categories, with the user’s most commonly used 30 emoji appearing at the front of the sidescrolling list. Certain notionally human emoji may be presented with 5 different skin tones, annotated as I-II, III, IV, V, and yellow, based on a modified version of the Fitzpatrick Scale used in dermatology.^13^ Skin-tone modification is accomplished practically by holding down on the emoji in the palette and selecting from a pop-up of skin-tone options. The I-II modifier represents the lightest skin tone, and the V modifier represents the darkest. Likewise, certain notionally human emoji can be modified in the same way for gender presentation, as either male, female, or nonbinary. While some keyboards automatically convert emoticons into emoji (for instance, entering the sequence :-) may automatically generate 😊), our CTS did not do so.

### Data Collection

We analyzed all clinical text messages involving hospitalists (physicians and advanced practice clinicians) of the medical center. Hospitalists were chosen because their inpatient workflow required frequent use of the CTS. We reviewed a 1% random sample of threads to and from hospitalists during our study period from July 2020 until March 2021, including messages sent by non-hospitalists to hospitalists and messages from hospitalists to other clinicians. Message threads are one or more messages sent between users as part of a closed conversation. The mean thread length was 5 messages. All messages were deidentified so that the study team did not know who the individual participants were.

We numbered threads in the full corpus of hospitalist messages provided by Diagnotes, and then used a random number generator with no repeats to select message threads to include in our sample. Given the exploratory nature of this project, the sample size was not determined by the need to test a hypothesis; rather, this 1% sample allowed our manual analysis to reach thematic saturation.^14,15^ Saturation was determined when no new insights regarding our stated questions related to clinicians’ use of ideograms emerged from additional data collection, with new data being too similar to old data to aid in further categorization. In qualitative research, saturation is held to be a best practice for determining sample size.^16^ We also drew on available internal, self-reported demographic data to characterize the hospitalists.

### Content Analysis

Our content analysis occurred in 2 rounds. First, we reviewed all threads in the sample for the presence or absence of an ideogram. This manual analysis allowed team members to assess ideograms in the rich and unfolding interactional context. It also allowed us to ensure that character sequences coded as emoticons did indeed represent emoticons. Next, for threads that contained at least 1 ideogram, we identified the specific type of ideogram used, and its associated features (eg, the skin tone of the emoji, whether it was a face or an object).

We then evaluated the communicative function served by the ideogram by examining what semantic and/or pragmatic contribution it made to the text and ongoing interaction within the full thread. Our coding was adapted from Roman Jakobson’s classic functions of language classification, defined through inclusion and exclusion criteria derived from his definitions of each function.^17^ Confusion and/or disagreement regarding assigned codes were resolved through consensus, and ideograms could be assigned more than one function code. Consensus discussions allowed for reflexive awareness in our interpretation, and the rich contextualization of stretches of text within their larger interactions allowed for a fuller understanding of the ideograms’ effects on the unfolding communication.

We assigned numerical pseudonyms to the deidentified users, allowing us to track texting practice of individual clinicians within and across threads. We summarized the frequency of ideogram use and patterns of deployment by individual users by means of descriptive statistics. We also collected data on any commentary related to the interactants’ uptake of ideograms as such, including their perceptions of appropriateness and acceptability.

## Results

During the study period, a total of 129 360 message threads (groups of individual messages functioning as part of a conversation between users) were sent to and from 80 unique hospitalists (49 [61%] were male; 30 [37%] were Asian, 5 [6%] were Black or African American, 2 [3%] were Hispanic or Latinx, 42 [53%] were White). Of the total threads sent, we analyzed 1319 threads, representing a 1% sample. Of the 41 hospitalists with age data, most were younger than 45 years of age (13 [32%] were aged 25-34 years, 19 [46%] were aged 35-44 years), and on average, they had spent 13 years in clinical practice. Table 1 describes their demographic characteristics. The demographic data were drawn from 2 internal departmental sources, resulting in the 2 sources having slightly different sums.

**Table 1.** Hospitalist Characteristics

Characteristic	Hospitalists, No. (%)
Sex	
Male	49 (61)
Female	31 (39)
Race and ethnicity	
Asian	30 (37)
Black or African American	5 (6)
Hispanic or Latinx	2 (3)
White	42 (53)
≥2 Races	1 (1)
Age, y	
25-34	13 (32)
35-44	19 (46)
45-54	4 (10)
55-64	5 (12)

**Figure 1.**
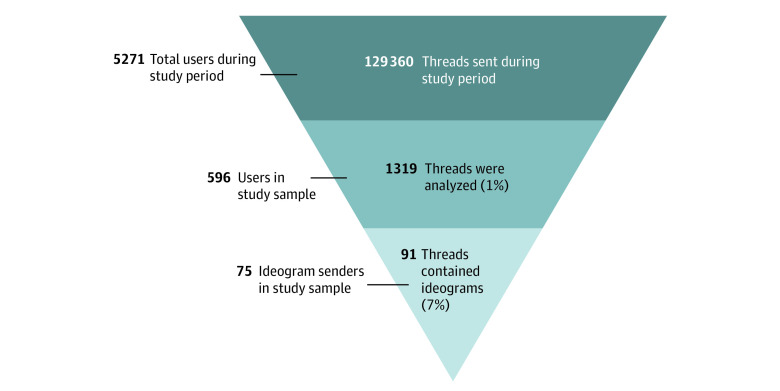
Sample Breakdown

In the 1319 threads analyzed, we identified 91 (7%) that contained at least 1 ideogram, for a total of 155 total individual messages with an ideogram. Both emoji and emoticons were used in messages sent between CTS users. Of a total of 596 unique users in our sample, 75 (13%) sent messages that contained at least 1 ideogram. (See Figure 1 for a visualization of our sample breakdown.)

### Commonly Used Emoji and Emoticons

**Figure 2.**
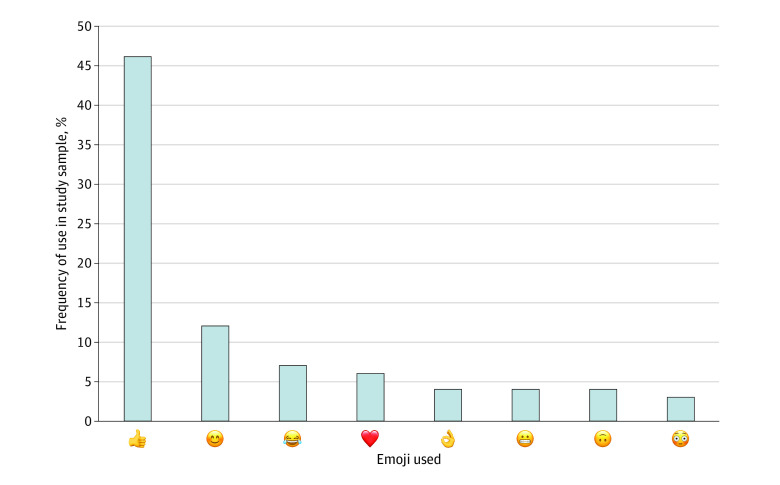
Frequency of the 8 Most Commonly Used Emoji by Hospitalists in Clinical Text Messages

Forty-two different types of emoji were used within these messages, and 11 messages (9%) contained more than 1 emoji. The most commonly used emoji included the thumbs-up (👍; 46 [39%]), the smiley face (😊; 10 [9%]), the tears of joy face (😂; 17 [6%]), and the heart (❤️; 6 [5%]). (See Figure 2 for additional information about emoji use results.)

Fifty-three emoji (43%) in our data set were modifiable for skin tone or gender. Of the 59 emoji modifiable for skin tone, 31 (53%) used such a modifier. Thirteen emoji (42%) were modified with the lightest skin tone I-II, 6 (19%) were modified with skin tone III, 10 (32%) were modified with skin tone IV, and 2 (6%) were modified with skin tone V. For gender, 8 emoji were modified out of a total 9 potentially modifiable emoji (89%). Six emoji (75%) were modified as female, and 2 (25%) were modified as male.

Overall, 38 messages (3%) contained emoticons. Users thus deployed emoji 3 times more often than emoticons. Four different types of emoticons were present in our data set, namely, :), :(, :/, and :-). No messages contained more than 1 emoticon. The most commonly used emoticon was :) (29 [73%]), followed by :( (5 [13%]), :/ (13 [8%]), and :-) (3 [8%]). While several users (6 [8%]) deployed a combination of emoji and emoticons in their messages, most users deployed either solely emoji (55 [73%]) or solely emoticons (14 [19%]).

### Functions of Emoji and Emoticons

Emoji served a number of linguistic functions in the messages in which they occurred. We present definitions, examples, and prevalence of ideogram functionality in Table 2. The most common use of ideograms was for emotive effect (94 [61%]), often to communicate information about the internal state of the sender or to assist in the recipient’s uptake of the message by qualifying its interpretation. An example message included, “just add flagyl for anaerobes and ditch the clinda! :).” Ideograms were additionally often used phatically (49 [32%]) as a means to open, maintain, close, or acknowledge a channel of communication.

**Table 2.** Communicative Functions of Emoji and Emoticons^a^

Term	Definition	Example	No. (%)
Emotive	Conveys information about the internal state of the sender; can also qualify the interpretation of a message	“I will strike while the iron is hot...after lunch of course 😂”	94 (61)
Phatic	Opens, maintains, or closes the channel of communication; often marks acknowledgment of the preceding message	“Ok thanks. I’ll ask the unit to give him a copy once you’re able to sign.” / “👍”	49 (32)
Conative	Conveys information about (or performatively manages) the internal state of the recipient; often serves to soften requests and “requestions”^16^ as an innovative politeness strategy	“Please let me know if you have any questions.:)”	13 (8)
Referential	Adds or duplicates at-issue propositional information in the message	“Meaning go ahead and change the order right now right?” / “Yes 👍”	11 (7)
Poetic	Structures the message visually	“HAPPY BIRTHDAY 🎁 🎈 🎂”	2 (1)

^a^ This table’s definitions were adapted from Jakobson,^17^ 1960.

Of the ideograms with an emotive function, 69% conveyed a positive affect, such as happiness or good humor, while only 31% conveyed a negative affect, such as sorrow or disappointment. Only a single token accompanied a message with negative affect directed at the addressee, and in this instance, the emoji functioned to soften the impact of the sender’s criticism of the addressee: “You abandoned me! […] 🤪 that was mildly painful.” All other negative emotivity was commiserative and thus could be seen as relationship building or affirming. For instance, sorrow conveyed through ideograms was often coupled with apologies: “Sorry about the direct from hell 😊.”

The emotive addition of ideograms substantially altered the interpretation of certain messages. One can observe, for example, the following exchange:

User 928: “You following [patient name], today?”

User 070: “Yes I have the pleasure of her today 🙃”

User 928: “Oh how fun 😝”

Here, the emoji reframed the message thread as sarcastic rather than sincere, recontextualizing the information and interactional dynamics it conveys.

Clinicians rarely used emoji to refer to objects, events, or ideas (11 [7% of tokens]). However, ideogram use was largely adjunctive, adding new information to the messages in most instances (22 [71%]), with only 33 (29%) of the ideograms solely duplicating information that was already present in the natural language portion of the message. Table 3 summarizes the additional features of ideogram use. By and large, these ideograms were not used as mere visual flair as a part of the poetic function (2 tokens [2% of tokens]). Neither were they frequently used in a playful, substitutive manner, in which they simply took the place of natural language lexical items (1 token [1% of tokens]).

**Table 3.** Additional Features of Emoji and Emoticon Use

Term	Definition	Example	No. (%)
Duplication	Does not add new information but rather duplicates already-present information (rather than substitutes for it)	“Awesome 👍🏽”	33 (29)
Emphasis	Emphasizes an already-existing feature (eg, emotive force) of a message but does not duplicate it completely	“Thank you:)”	13 (8)
Reduplication	Repeats 1 emoji type multiple times, typically as a strategy for emphasis	“I’m a lover not a fighter…” / “🤣🤣🤣”	7 (6)
Substitution	Replaces a natural-language lexical item, typically playfully	“Ur 🙅🏻﻿♂️ boss of 🧔🏻♂️”	1 (1)

## Discussion

### Emoji and Emoticon Use

In our analysis of the use of emoji and emoticons in text messages between clinicians, we found that these ideograms perform a variety of communicative functions. While clinicians rarely used ideograms to refer to objects, events, or ideas (11 [7% of tokens]), these symbols nonetheless added at least some new information in the majority of instances (122 [71%]). Clinicians used ideograms most often to add emotive content to their texts (61%). In particular, they modulated the intensity of speech acts,^18^ as in the message “just add flagyl for anaerobes and ditch the clinda! :).” Because emotions are often conveyed through features of language not representable in standard orthographic format, the addition of this information through ideograms can provide important contextualization, evaluation, and disambiguation (explained later) for the messages to which they are added.^19,20,21^

While some tokens in our sample were clearly intended to be humorous in the emotive information they conveyed, others had a more practical purpose. In fact, in some cases the emotive addition of ideograms substantially altered the interpretation of messages, such as by reframing the message thread as sarcastic rather than sincere. This disambiguation is a particularly useful affordance in textual health care communication, where ambiguity is notoriously high,^22^ as is the need for appropriate affect maintenance in the midst of emotionally demanding situations.^23^ The use of these ideograms may also change how the thread could be interpreted if it were read by a third party, for instance, a supervisor, a court, or the patient him- or herself, though it remains unclear in what precise way. All utterances may be taken up in different ways by different parties, and in the clinical context, an utterance’s receipt by unintended interlocutors may have consequences beyond the scope of the current analysis.

Clinicians in our sample also regularly used ideograms phatically (32%), by which we mean that they opened, maintained, or closed a channel of communication. In fact, the most commonly used emoji (the thumbs-up or 👍) constituted 39% of all emoji in our sample and was often used in similar fashion to the thumbs-up tapback button available in our study’s CTS interface for recipients to react to messages. Phatic uses signaled that a message had been received or that a plan of action had been acknowledged. This usage is notable, as one study found that some clinicians are frustrated by receiving phatic messages along the lines of “OK thanks,” believing the additional message to be a superfluity and a burden on their time.^24^ However, such phatic acknowledgment may also serve the clinically relevant function of confirming a shared understanding of a given medical situation.

Moreover, the phatic use of ideograms may also soften the tone or increase the politeness of a message. It is in this way that ideograms have the potential to manage and even improve interpersonal relationships and promote positive interactions in the setting of text messaging as the de facto alternative to voice-based communication.^20,25,26^ In fact, ideograms in our study tended to be used to convey positive rather than negative affect, affirming the results from another study.^27^ Our findings also echo those of a focus group on user experiences with clinical texting. In that study, Lee and colleagues^24^ discovered that while some users complained about the use of emoji in texts from nurses, others defended it as a way of conveying positive affect. For instance, one participant stated, “The emoji allows there to be another layer of personal interaction.” In our study, even ideograms that conveyed negative affect typically did so in a way that could serve to improve or reinforce the sender’s relationship with the addressee, as has been found in others studies.^26,28^ For instance, many of these ideograms were used to apologize or to commiserate, thereby potentially working to repair interpersonal dynamics. And unlike emotion-bearing elements of spoken or signed language, emoji are always voluntarily selected, allowing for the affect they convey to be more consciously and conscientiously encoded.

### Professionalism and Appropriateness

Both popular media and institutional policies have suggested that the use of ideograms in professional communication may be inappropriate. Some studies have likewise found ambiguity in their reception. For instance, Riordan and Glikson^29^ found that while male respondents rated workplace leaders’ likability and effectiveness higher when their correspondence included emoji, women rated these leaders as less effective and less appropriate. Glikson and colleagues^30^ separately found that the use of the smiley (😊) could decrease the perception of competence in work-related contexts as it was seen as inappropriate for such a formal setting. Most strikingly, a study from 15 years ago suggested that the use of emoji could make the sender appear “childish.”^31^ Critically, however, although it was not the primary focus of our project to evaluate the professionalism of clinicians’ use of ideograms, our analysis did not identify their use as particularly disparaging of patients or triggering of medicolegal concerns. Nor did we observe any exchanges related to misunderstanding or other negative repercussions from their use. Published concerns about unprofessionalism, then, may stem from anticipated rather than observed issues.

Moreover, attitudes toward language use evolve over time, and public critiques of emoji use do not necessarily reflect the uptake and interpretation of emoji in practice.^32^ Scholars have recently begun to call for the incorporation of emoji into professional patient-clinician and public health communication.^5,6,8,10^ While we are unaware of any empirical publications regarding the use of emoji in professional communication between medical colleagues, many of the considerations in the extant literature seem relevant to our case as well: emoji may enhance social relationships by better communicating sender’s emotions.^25^ The complexity and challenges of team communication in health care are well documented, both in studies that focus on specific relationship dynamics (eg, physician-nursing communication) and those whose focus is on a specific clinical process (eg, communication around handoffs).^33,34,35^ The phatic and emotive functions of emoji, as we observed in our data, may serve to facilitate team communication by efficiently conveying positive affect and agreement.

Emoji have become a commonplace in computer-mediated communication over the last 2 decades. One in 5 posts on the microblogging platform Twitter contains at least 1 emoji,^36^ nearly half of all Instagram messages contain emoji,^37^ and 92% of the online population have used emoji in their communications.^38^ The appearance of ideograms in clinical texting, then, may not be surprising. More importantly, we have found no reason to characterize their use as professionally worrisome. They appear primarily to be used to add new, interactionally salient information to communication; to convey primarily positive affect; and to have the potential to improve interpersonal dynamics. However, more research is needed to examine the perception and burden of phatic uses of ideograms and to determine best practices.

### Limitations

This study had some limitations. Our data focused on the clinical text messages that were either sent by or sent to hospitalists at a single academic medical center. Although the hospitalists in our sample communicated with many different team members across the health system—including social workers, surgeons, hospital administrators, and many others—our understanding of clinicians’ use of emoji and emoticons is limited to these users. Clinicians in different health systems or settings may have their own norms and practices. Yet while our analysis may have a somewhat limited breadth, we used a novel source of data—that of CTSs—to provide an in-depth analysis of the different communicative functions of emoji and emoticons used by clinicians. Another limitation is the Jakobson^17^ framework was initially developed for spoken language. However, it has been applied to computer-mediated communication by other researchers with success.^39^

## Conclusion

Our study found that clinicians typically use emoji and emoticons to add new information, to disambiguate affect, and to promote interpersonal relationships. While not standard in traditional English orthography, in our content analysis of ideogram use in CTS, we did not find a reason to consider their use as different from other, acceptable tone-modulating devices, such as capitalization or the use of nonstandard punctuation (eg, multiple exclamation marks). These results may suggest that concerns about emoji’s and emoticons’ adverse effect on professionalism may be overstated. Further research is warranted in the area of emoji and emoticon use among clinicians, including in other forms of computer-mediated communication or using other methods of analysis, such as a natural language processing approach.

## Notes

Supplement.
Data Sharing Statement

## References

[1] Pavalanathan U, Eisenstein J. More emojis, less:) the competition for paralinguistic function in microblog writing. First Monday. Published online October 20, 2016. doi:10.5210/fm.v21i11.6879

[2] Emogi Research Team. 2015 Emoji Report. 2015. Accessed May 12, 2023. https://www.almendron.com/tribuna/wp-content/uploads/2016/08/Emoji_Report_2015.pdf

[3] Krohn FB. A generational approach to using emoticons as nonverbal communication. J Tech Writ Commun. 2004;34(4):321-328. doi:10.2190/9EQH-DE81-CWG1-QLL9

[4] Loglia JM, Bowers CA. Emoticons in Business Communication. In: Tettegah S, Noble S, eds. Emotions, Technology, and Design. Elsevier; 2016:37-53. doi:10.1016/B978-0-12-801872-9.00003-X

[5] Boender TS, Louis-Ferdinand N, Duschek G. Digital visual communication for public health: design proposal for a vaccinated emoji. J Med Internet Res. 2022;24(4):e35786. doi:10.2196/35786 35389363 PMC8993141

[6] He S, Renne A, Argandykov D, Convissar D, Lee J. Comparison of an emoji-based visual analog scale with a numeric rating scale for pain assessment. JAMA. 2022;328(2):208-209. doi:10.1001/jama.2022.7489 35819433 PMC9277495

[7] Pourmand A, Quan T, Amini SB, Sikka N. Can emoji’s assess patients’ mood and emotion in the emergency department? an emoji based study. Am J Emerg Med. 2020;38(4):842-843. doi:10.1016/j.ajem.2019.09.008 31761435

[8] Setty JV, Srinivasan I, Radhakrishna S, Melwani AM, Dr MK. Use of an animated emoji scale as a novel tool for anxiety assessment in children. J Dent Anesth Pain Med. 2019;19(4):227-233. doi:10.17245/jdapm.2019.19.4.227 31501781 PMC6726885

[9] Szeto MD, Barber C, Ranpariya VK, . Emojis and emoticons in health care and dermatology communication: narrative review. JMIR Dermatol. 2022;5(3):e33851. doi:10.2196/33851 36405493 PMC9642845

[10] Lai D, Lee J, He S. Emoji for the medical community-challenges and opportunities. JAMA. 2021;326(9):795-796. doi:10.1001/jama.2021.8409 34547099

[11] Wicks P. A millennial discharge summary. BMJ. Published online December 15, 2016. doi:10.1136/bmj.i6607

[12] O’Reilly-Shah VN, Lynde GC, Jabaley CS. Is it time to start using the emoji in biomedical literature? BMJ. Published online December 12, 2018. doi:10.1136/bmj.k5033

[13] Fitzpatrick TB. The validity and practicality of sun-reactive skin types I through VI. Arch Dermatol. 1988;124(6):869-871. doi:10.1001/archderm.1988.01670060015008 3377516

[14] Charmaz K. Theoretical sampling, saturation, and sorting. In: Constructing Grounded Theory. Sage Publications Inc; 2014:192-224.

[15] Corbin J, Strauss A. Basics of Qualitative Research: Techniques and Procedures for Developing Grounded Theory. 3rd ed. Sage Publications Inc; 2008. doi:10.4135/9781452230153

[16] Guest G. How many interviews are enough?: an experiment with data saturation and variability. Field Methods. 2006;18(1):59-82. doi:10.1177/1525822X05279903

[17] Jakobson R. Linguistics and poetics. In: Sebeok TA, ed. Style in Language. MIT Press; 1960:350-377.

[18] Sampietro A. Emoji and rapport management in Spanish WhatsApp chats. J Pragmatics. 2019;143:109-120. doi:10.1016/j.pragma.2019.02.009

[19] Al Rashdi F. Functions of emojis in WhatsApp interaction among Omanis. Discourse Context Media. 2018;26:117-126. doi:10.1016/j.dcm.2018.07.001

[20] Gibson W, Huang P, Yu Q. Emoji and communicative action: the semiotics, sequence and gestural actions of ‘face covering hand’. Discourse Context Media. 2018;26:91-99. doi:10.1016/j.dcm.2018.05.005

[21] Kaye LK, Wall HJ, Malone SA. “Turn that frown upside-down”: a contextual account of emoticon usage on different virtual platforms. Comput Human Behav. 2016;60:463-467. doi:10.1016/j.chb.2016.02.088

[22] Lee JL, Matthias MS, Huffman M, Frankel RM, Weiner M. Insecure messaging: how clinicians approach potentially problematic messages from patients. JAMIA Open. 2020;3(4):576-582. doi:10.1093/jamiaopen/ooaa051 33758796 PMC7969962

[23] Roter DL, Frankel RM, Hall JA, Sluyter D. The expression of emotion through nonverbal behavior in medical visits. mechanisms and outcomes. J Gen Intern Med. 2006;21(Suppl 1)(suppl 1):S28-S34. doi:10.1111/j.1525-1497.2006.00306.x 16405706 PMC1484830

[24] Lee JL, Kara A, Huffman M, . Qualitative analysis of team communication with a clinical texting system at a midwestern academic hospital. Appl Clin Inform. 2022;13(2):391-397. doi:10.1055/s-0042-1744389 35294986 PMC8926456

[25] Riordan MA. Emojis as tools for emotion work: communicating affect in text messages. J Lang Soc Psychol. 2017;36(5):549-567. doi:10.1177/0261927X17704238

[26] Albawardi A. The translingual digital practices of Saudi females on WhatsApp. Discourse Context Media. 2018;25:68-77. doi:10.1016/j.dcm.2018.03.009

[27] Cheng L. ¿Digo lo que siento y siento lo que digo? Una aproximación transcultural al uso de los emoticonos y emojis en los mensajes en CMC. Fonseca. J Commun. 2017;15(15):199. doi:10.14201/fjc201715199217

[28] Gesselman AN, Ta VP, Garcia JR. Worth a thousand interpersonal words: emoji as affective signals for relationship-oriented digital communication. PLoS ONE. 2019;14(8):e0221297. doi:10.1371/journal.pone.0221297 31415640 PMC6695182

[29] Riordan MA, Glikson E. On the hazards of the technology age: how using emojis affects perceptions of leaders. Int J Bus Commun. Published online November 24, 2020. doi:10.1177/2329488420971690

[30] Glikson E, Cheshin A, van Kleef GA. The dark side of a smiley: effects of smiling emoticons on virtual first impressions. Soc Psychol Personal Sci. 2018;9(5):614-625. doi:10.1177/1948550617720269

[31] Provine RR, Spencer RJ, Mandell DL. Emotional expression online: emoticons punctuate website text messages. J Lang Soc Psychol. 2007;26(3):299-307. doi:10.1177/0261927X06303481

[32] Halverson CME. Skin-tone modified emoji and first-person indexicality. Soc Semiot. Published online November 24, 2021. doi:10.1080/10350330.2021.2000333

[33] Manojlovich M, Harrod M, Hofer T, Lafferty M, McBratnie M, Krein SL. Factors influencing physician responsiveness to nurse-initiated communication: a qualitative study. BMJ Qual Saf. 2021;30(9):747-754. doi:10.1136/bmjqs-2020-011441 33168635 PMC8140397

[34] Matziou V, Vlahioti E, Perdikaris P, Matziou T, Megapanou E, Petsios K. Physician and nursing perceptions concerning interprofessional communication and collaboration. J Interprof Care. 2014;28(6):526-533. doi:10.3109/13561820.2014.934338 25003547

[35] House S, Havens D; Nurses’ and Physicians’ Perceptions of Nurse-Physician Collaboration. Nurses’ and physicians’ perceptions of nurse-physician collaboration: a systematic review. J Nurs Adm. 2017;47(3):165-171. doi:10.1097/NNA.0000000000000460 28157818

[36] Broni K. Emoji trends that defined 2020. Emojipedia. Published December 29, 2020. Accessed May 10, 2023. https://blog.emojipedia.org/emoji-trends-that-defined-2020/

[37] Dimson T. Emojineering Part 1: Machine Learning for Emoji Trends. All Things Linguistic. 2015. Accessed May 12, 2023. https://allthingslinguistic.com/post/124609017512/emojineering-part-1-machine-learning-for-emoji

[38] Emogi Research Team. 2016 Emoji Report. Published 2016. Accessed May 12, 2023. https://destinationthink.com/wp-content/uploads/2018/10/2016_emoji_report.pdf

[39] Alshboul N, Rababah L. The emoji linguistic functions on Facebook interactions among undergraduate students at Jadara University in Jordan. JSEL. 2021;9(1):43. doi:10.5296/jsel.v9i1.18486

