# Supplementary material for: Content Analysis of Emoji and Emoticon Use in Clinical Texting Systems
Source: JAMA Netw Open. 2023 Jun 13;6(6):e2318140. doi: 10.1001/jamanetworkopen.2023.18140 (PMC10265294; doi:10.1001/jamanetworkopen.2023.18140)
Supplement: Supplement. — Data Sharing Statement [file jamanetwopen-e2318140-s001.pdf]

Data Sharing Statement

Halverson. Content Analysis of Emoji and Emoticon Use in Clinical Texting Systems. *JAMA Netw Open*. Published June 13, 2023. doi:10.1001/jamanetworkopen.2023.18140

Data
Data available: No
